# Lung microRNA deregulation associated with impaired alveolarization in rats after intrauterine growth restriction

**DOI:** 10.1371/journal.pone.0190445

**Published:** 2017-12-29

**Authors:** Pauline Dravet-Gounot, Cécile Morin, Sébastien Jacques, Florent Dumont, Fabiola Ely-Marius, Daniel Vaiman, Pierre-Henri Jarreau, Céline Méhats, Elodie Zana-Taïeb

**Affiliations:** 1 Inserm, U1016, Institut Cochin, Paris, France; 2 CNRS, UMR8104, Paris, France; 3 Université Paris Descartes, Faculté de Médecine, Paris, France; 4 AP-HP, Maternité Port Royal, Service de Médecine et Réanimation Néonatales, Paris, France; 5 DHU Risques et grossesse, Maternité Port-Royal, Paris, France; 6 Inserm U1141, Paris, France; 7 Premup, Paris, France; Institut de Pharmacologie Moleculaire et Cellulaire, FRANCE

## Abstract

Intrauterine growth restriction (IUGR) was recently described as an independent risk factor of bronchopulmonary dysplasia, the main respiratory sequelae of preterm birth. We previously showed impaired alveolarization in rat pups born with IUGR induced by a low-protein diet (LPD) during gestation. We conducted a genome-wide analysis of gene expression and found the involvement of several pathways such as cell adhesion. Here, we describe our unbiased microRNA (miRNA) profiling by microarray assay and validation by qPCR at postnatal days 10 and 21 (P10 and P21) in lungs of rat pups with LPD-induced lung-alveolarization disorder after IUGR. We identified 13 miRNAs with more than two-fold differential expression between control lungs and LPD-induced IUGR lungs. Validated and predicted target genes of these miRNAs were related to “tissue repair” at P10 and “cellular communication regulation” at P21. We predicted the deregulation of several genes associated with these pathways. Especially, E2F3, a transcription factor involved in cell cycle control, was expressed in developing alveoli, and its mRNA and protein levels were significantly increased at P21 after IUGR. Hence, IUGR affects the expression of selected miRNAs during lung alveolarization. These results provide a basis for deciphering the mechanistic contributions of IUGR to impaired alveolarization.

## Introduction

Bronchopulmonary dysplasia (BPD) is the main respiratory sequelae of preterm birth [1]. BPD is characterized by arrested alveolar development, with fewer but larger alveoli and impaired capillaries, leading to oxygen dependency, prolonged hospital stay, and increased risk of hospital readmission in early infancy [1, 2]. The mechanisms leading to abnormal alveolarization are not fully elucidated. Multiple factors contribute to BPD pathogenesis in the pre- and/or postnatal period, considered aggression in immature lungs, including oxygen toxicity, baro- and volo-trauma, and bacterial infection. Alone, intrauterine growth restriction (IUGR) multiplies by 6 the risk of BPD, although the pathogenesis is unclear [3].

We previously explored a model of IUGR induced by a low-protein diet (LPD) given to rat dams during gestation. The treatment triggers sustained alveolarization disorder in rat pups. Intriguingly, none of the factors previously described as essential for alveolarization were found modified in this model [4]. Genome-wide analysis before, during, and at the end of the alveolarization process highlighted that LPD-induced IUGR disturbed mainly pathways associated with “cell adhesion”, “molecular adhesion and antigens” and “peroxisome proliferator-activated receptor” in lungs. The related genes appeared to be organized as a network, which suggested upstream molecular regulation.

MicroRNAs (miRNAs) are a class of small, non-coding, highly stable RNAs that have important and complex epigenetic roles in regulating mRNA and protein expression in various physiological and pathological processes [5]. Compelling evidence suggests the role of miRNAs, especially in cancer biology, having potential as diagnostic, prognostic and predictive biomarkers [6]. One miRNA can target multiple mRNAs and one mRNA can be the target of multiple miRNAs, defining gene expression networks.

Specific miRNAs present dynamic expression changes during lung development [7]. Deregulation of miRNA expression has been found in chorioamnionitis- and hyperoxia-impaired lung alveolarization [8–12]. Here, we profiled the global miRNA expression in rat pup lungs in a LPD-induced IUGR model at two key times during alveolarization: postnatal day 10 (P10) and day 21 (P21). The results of both miRNA and gene expression profiles in this BPD model were further integrated to highlight new pathways and deregulated gene networks related to IUGR-impaired alveolarization.

## Materials and methods

### Animals and diet

The animal model was described previously [4]. Briefly, Sprague-Dawley rat dams, housed in Charles River facilities (l’Arbresle, France), were randomly divided into two groups by protein diet during gestation: 9% LPD versus 23% control diet. LPD was isocaloric by increasing the carbohydrates ratio. Diets were given to dams from the day of conception until postnatal day 2 (P2). Postnatal day 0 (P0) was defined by the day of birth. To avoid extrauterine growth restriction, litters were equalized at P2 to 10 pups. Rat pups were killed at P10 and P21 by intraperitoneal overdose of pentobarbital sodium (70 μg/g body weight) and then exsanguinated by aortic transection; lungs were harvested and frozen. At P10, 2 males and 3 females were analyzed in control diet during gestation and LPD-induced IUGR groups. At P21, 3 males and 2 females were analyzed in the control group and 2 males and 3 females in the LPD-induced IUGR group. All experiments were approved by INSERM ethical rules, and the US National Research Council’s Guide for the Care and Use of Laboratory Animals was followed. The Charles River Laboratories Ethics Committee approved the LPD protocol (10/17/11 and 07/28/12).

### RNA extraction and preparation and microarray hybridization

Total RNA was extracted from the entire lung tissue of 20 rat pups, 10 at P10 and 10 at P21, by using Trizol (Invitrogen, Carlsbad, CA) as the manufacturer instructed. Purity and concentration of total RNA samples were first evaluated by using a Nanodrop spectrophotometer (Thermo Scientific, Waltham, MA), by measuring absorbance at 260 nm, and samples were run in a RNA nano-chip by using the Bioanalyzer System (Agilent Technologies, Santa Clara, CA) to verify their integrity. All samples with RNA integrity score > 6.5 were stored at -80°C until processing.

The cRNA labeling and hybridization involved use of miRNA 4.0 Affymetrix GeneChips (Affymetrix, Santa Clara, CA) according to protocols from Affymetrix in the Genom’IC platform (Institut Cochin, Paris). After overnight hybridization, the miRNA 4.0 chips were washed and scanned by using the GCS3000 7G. The scanned images were then analyzed by using Expression Console software (Affymetrix) to obtain raw data (CEL files) and metrics for quality control. No apparent outliers were detected.

### Microarray analysis

Specific miRNA analysis involved use of Partek Flow v3.0 (Partek Inc., St. Louis, MO). CEL files were imported and normalized by Robust Multi-array Averaging (RMA). The miRNA 4.0 chip contained probes for 490 rat pre-miRNAs and 728 rat mature miRNAs. miRNAs were selected according their average expression levels. miRNAs with nominal p ≤ 0.05 between the two groups of protein diets during gestation were considered differentially expressed. Among these, mature miRNAs showing two-fold variation in expression were retained for further analysis. The complete dataset is available as a Gene Expression Omnibus (GEO) profile in the GEO database (www.ncbi.nih.gov/geo/; accession no. GSE80123).

Hierarchical clustering and heatmap analysis involved use of the MeV Package [13]. To estimate the biologic effects of differentially expressed miRNAs, lists of validated and predicted target genes were obtained from Tarbase and Mirtarbase. Gene ontology (GO) enrichment analysis involved use of Genomatix GePS (release 2.4.0, Genomatix BH, Munich).

### cDNA synthesis and real-time qPCR

For miRNA expression analysis, total RNA (1 μg) was converted to cDNA by using the qScript microRNA cDNA synthesis kit (Quanta Biosciences, Gaithersburg, MD) as instructed. For gene expression analysis, total RNA (2 μg) was converted to cDNA by using a homemade protocol with M-MLV transcriptase reverse (Invitrogen, Fisher Scientific, Illkirch, France) [14].

Quantitative PCR involved use of the Light Cycler 480, 96-well apparatus (Roche Diagnostics, Manheim, Germany). Briefly, Perfecta microRNA Assays (Quanta Biosciences) were used to quantify miRNAs, with use of the Light Cycler 480 SYBR Green I Master kit (Roche). Primers for gene expression were chosen by using PRIMER3 software, based on published sequences (Table 1). The amplifications were analyzed by the LightCycler software using the three fit points method. The PCR efficiency was experimentally assessed and was above 90% for all the amplifications. All measurements were performed in duplicate. The results are expressed as Ct values and normalized on the calculated median Ct of each sample (ΔCt). Relative expression was calculated using the comparative Ct method (2^−ΔΔCt^). For miRNA data normalization, miR-10a-3p and miR-124-3p were both used as endogenous controls because they present the least variability across the samples according to Normfinder [15]. For mRNA data normalization, Glyceraldehyde 3-phosphate dehydrogenase (GADPH) and matrix metalloproteinase 16 (MMP16) were our reference genes as previously used [16].

**Table 1:** Primer sequences used for RT-PCR.

Gene	Forward primer	Reverse primer
CTHRC1	GCTCAGCCAAATGAAAAAGC	GCATTCCGGTATATAGGCTCA
E2F3	TCCTACCTTCTTCCTCCAAGAG	GTCTGCAAGGAAAGCAGAGG
TNFSF9	TAGCCACCCTTCTTGTGACC	CAGCTGCCCAGCAGCAGTTACTA
IFNGR2	GAGTCCGCAGGAGACTTCAG	GGGAAGTCAAGCAAGAGTGG
NTRK3	GAACCACAACAGCGACTGAA	CGAAAGGAGTTGAGGTGAGG
MAP3K9	TCCTTGCACTGAAAATGCAC	TAAAGGCGAGGAAGACCAGA
GAPDH	TGATTCTACCCACGGCAAGTT	TGATGGGTTTCCCATTGATGA
MMP16	GAGCTGGGACATGCTCTAGG	GAGGGATCTTGTCAGGTGGA
miR	Primer	
miR-128-3p	UCACAGUGAACCGGUCUCUUU	
miR-34c-5p	AGGCAGUGUAGUUAGCUGAUUGC	
miR-434-3p	UUUGAACCAUCACUCGACUCCU	
miR-30e-5p	UGUAAACAUCCUUGACUGGAAG	
miR-23b-5p	GGGUUCCUGGCAUGCUGAUUU	
miR-451-5p	AAACCGUUACCAUUACUGAGUU	
miR-1839-5p	AAGGUAGAUAGAACAGGUCUUG	
miR-449a-5p	UGGCAGUGUAUUGUUAGCUGGU	
miR-19b-3p	UGUGCAAAUCCAUGCAAAACUGA	
miR-541-5p	AAAGGAUUCUGCUGUCGGUCCCACU	
miR-378a-5p	CUCCUGACUCCAGGUCCUGUGU	
miR-127-3p	UCGGAUCCGUCUGAGCUUGGCU	
miR-184	UGGACGGAGAACUGAUAAGGGU	
miR-10a-3p	CAAAUUCGUAUCUAGGGGAAUA	
miR-124-3p	UAAGGCACGCGGUGAAUGCC	

### Western blot analysis

Whole lungs were homogenized as previously described [16]. An amount of 30 μg protein was subjected to 12% SDS-PAGE and blotted onto nitrocellulose transfer membranes (Thermo Scientific), which were blocked in 0.1% Tris buffered saline-Tween 20 containing 5% nonfat milk. The membranes were incubated overnight with the primary polyclonal rabbit anti-E2F3 antibody (1:8,000; ab59917, Abcam, Cambridge, UK), then horseradish peroxidase-conjugated secondary antibodies (1:4,000; Pierce Antibodies) and visualized by using Supersignal WestPico Reagents (Thermo Scientific). Further labeling with beta-tubulin antibody (1:3,000; clone AA2, Millipore, Molsheim, France) was used as a loading control. Densitometry analysis of band intensities involved use of ImageJ v1.49a.

### Immunohistochemistry

Lung tissue sections 4 μm thick were deparaffinized in xylene and rehydrated through graded ethanol concentrations. After antigen retrieval in citrate buffer at pH 6.0 at 96°C, slides were processed by using the Polymer novolink kit (Leica, Nanterre, France). Slides were incubated overnight at 4°C with polyclonal anti-E2F3 antibody (1:800 in phosphate buffered saline-1% bovine serum albumin-0.1% Triton) and signals were developed with DAB used as a substrate. Signals were analyzed by using ImageJ to quantify the staining within the alveoli under a microscope equipped with a DC 300F camera (digital module R, IM 1000; Leica).

### Statistical analysis

All data were analyzed by using GraphPad Prism 6 (GraphPad Software, San Diego, CA). Unless indicated, we used non-parametric tests for unpaired samples. Pearson correlation coefficient was used for correlation analysis. P <0.05 was considered statistically significant.

## Results

### miRNA expression profile during alveolarization in rats after LPD-induced IUGR

**Fig 1:**
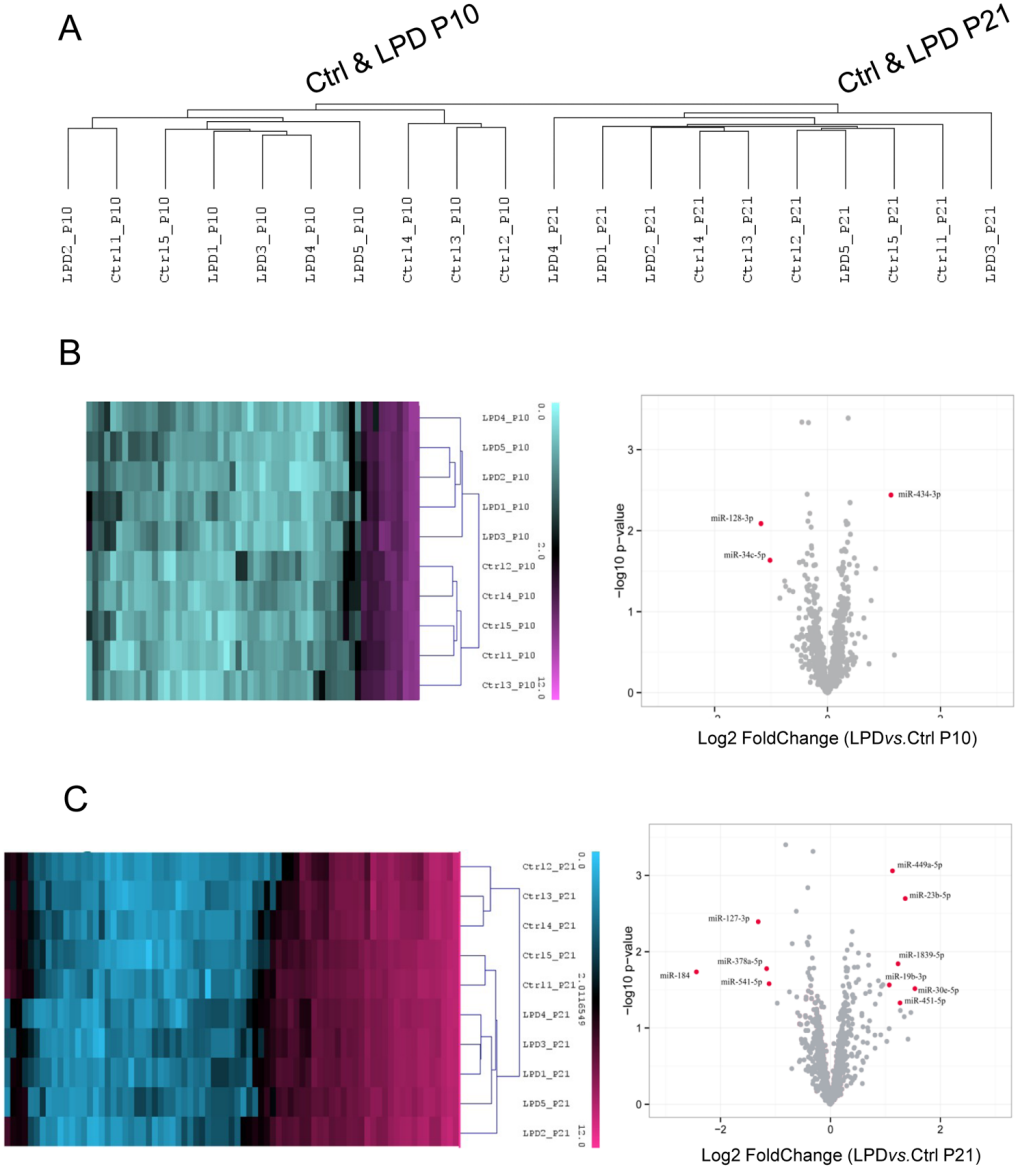
miRNA expression profiling in rat lungs at postnatal day 10 (P10), or during, and at the end (P21) of alveolarization after low-protein diet (LPD)-induced intrauterine growth restriction (IUGR). (A) Unsupervised hierarchical cluster analysis of miRNA expression in postnatal developing lungs after a control-diet gestation clearly distinguishes P21 from P10 samples. (B) Supervised hierarchical cluster analysis, heatmap representation (left panel) and volcano plot (right panel) of miRNAs differentially expressed at P10 after LPD-induced IUGR *vs*. control-diet gestation (*p* <0.05). (C) Supervised hierarchical cluster analysis, heatmap representation (left panel) and volcano plot (right panel) of miRNAs differentially expressed at P21 after LPD-induced IUGR *vs*. control-diet gestation (*p* <0.05). MiRNAs with significantly different expression between LPD-induced IUGR and control-diet gestation (fold-change ≥ 2; *p* < 0.05) are highlighted in red; miRNAs from total lungs of 5 animals/group were analyzed. We used qRT-PCR to validate this miRNA expression profile for the 13 differentially expressed miRNAs. We used the same rat pup samples than in the microarray experiments. The microarray and real-time PCR data were highly correlated, with r^2^ = 0.669 (*p* = 0.0003), thus inferring high reliability of the microarray data (Fig 2). Again the correlation was greater for P10 than P21 data. At P10, the differential expression of the three miRNAs was statistically confirmed, with miR128-3p and miR34c-5p downregulated and miR434-3p upregulated. At P21, the differential expression of 5 of 10 of the miRNAs was statistically confirmed, with miR378a-5p, miR127-3p, and miR184 downregulated and miR30e-5p and miR449a-5p upregulated.

Total RNA was obtained from five animals per group at P10 or P21 after LPD-induced IUGR or control diet during gestation. The 20 RNA samples (10 at each time) were hybridized independently with Affymetrix miRNA 4.0 arrays. Unsupervised hierarchical cluster analysis (Manhattan distance) of the 1218 probes corresponding to rat sequences (728 unique mature miRNAs) on the microarray resulted in a dendrogram with each time forming a distinct cluster (Fig 1). We then separately analyzed data for the two times. Supervised hierarchical clustering analysis at P10 revealed 56 mature miRNAs with differential expression at *p* < 0.05 between LPD-induced IUGR and control lungs (Fig 1). Among these miRNAs, three showed more than twofold differential expression (Fig 1): miR-128-3p and miR-34c-5p were significantly downregulated, and miR-434-3p was significantly upregulated. Analysis at P21 revealed 74 mature miRNAs with differential expression between LPD-induced IUGR rat lungs and control lungs (Fig 1): 10 showed more than twofold differential expression: miR-184, miR-127-3p, miR-378a-5p and miR541-5p were downregulated, and miR-30e-5p, miR-23b-5p, miR-451-5p, miR-1839-5p, miR-449a-5p, and miR-19b-3p were upregulated in LPD-induced IUGR versus control lungs. Detailed data of the 56 and 74 miRNAs modulated at P10 and P21 are summarized in S1 Table. Of note, at P10, the hierarchical clustering properly separated the two experimental groups, whereas at P21, two of the five control animals segregated more closely with the LPD-induced IUGR animals.

**Fig 2:**
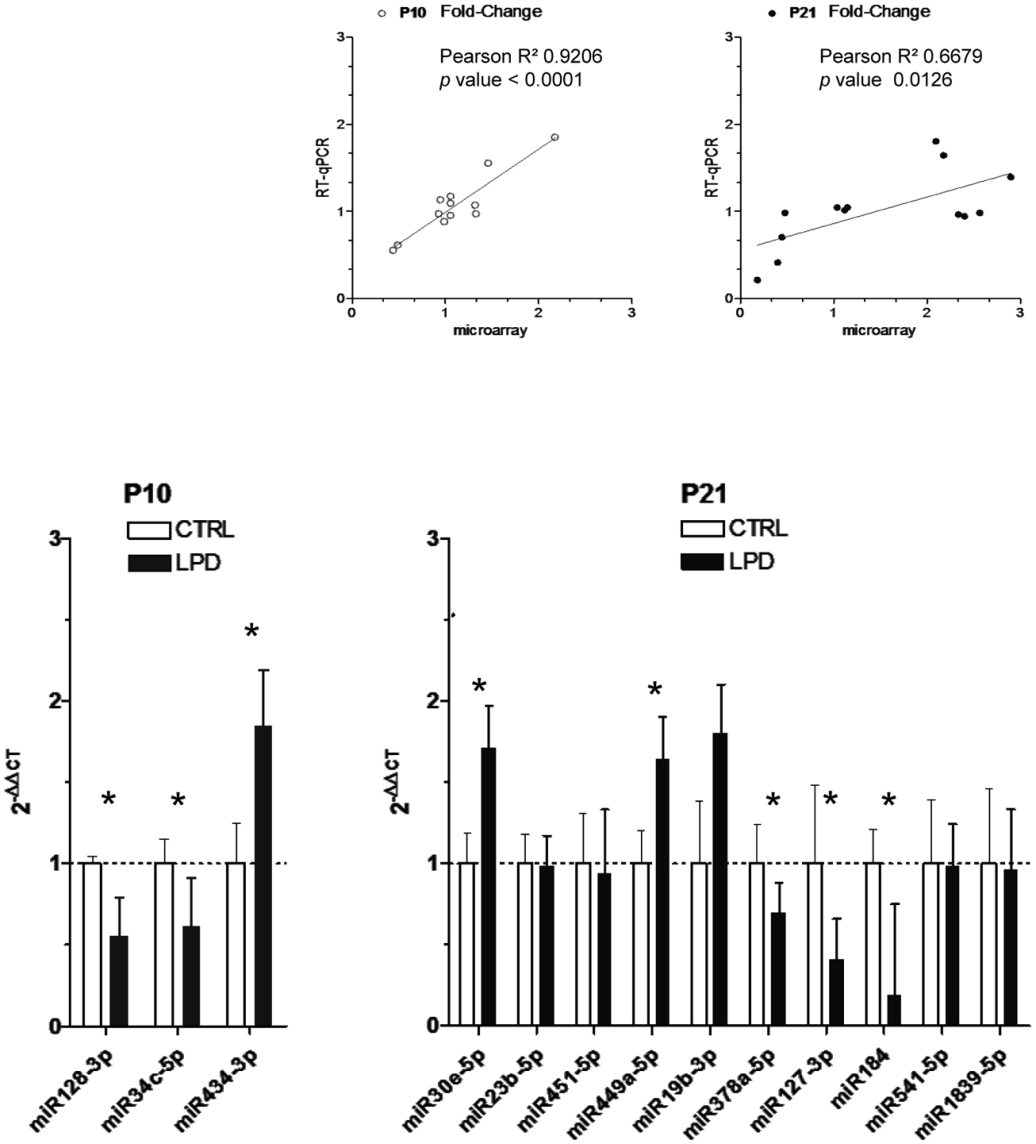
qRT-PCR analysis of differentially expressed miRNAs in rat lungs at P10 and P21 after LPD-induced IUGR. We confirmed the differential induction or repression detected by the global approach in 3 of 3 miRNAs at P10 after LPD-induced IUGR *vs*. control-diet gestation and 5 of 10 miRNAs at P21. The relative expression was normalized to that of miR-10a-3p and miR-124-3p, the two miRNAs showing the least variability across the samples according to Normfinder. Values are mean ± SEM for 5 animals/group. *, *p* < 0.05; two-tailed Mann-Whitney test. Insert: linear correlation between the fold-change of miRNA expression measured by qRT-PCR or microarray hybridization at the two times; Pearson r^2^ correlation value and *p* value are depicted.

### Target gene identification of differentially expressed miRNAs during alveolarization after LPD-induced IUGR

**Fig 3:**
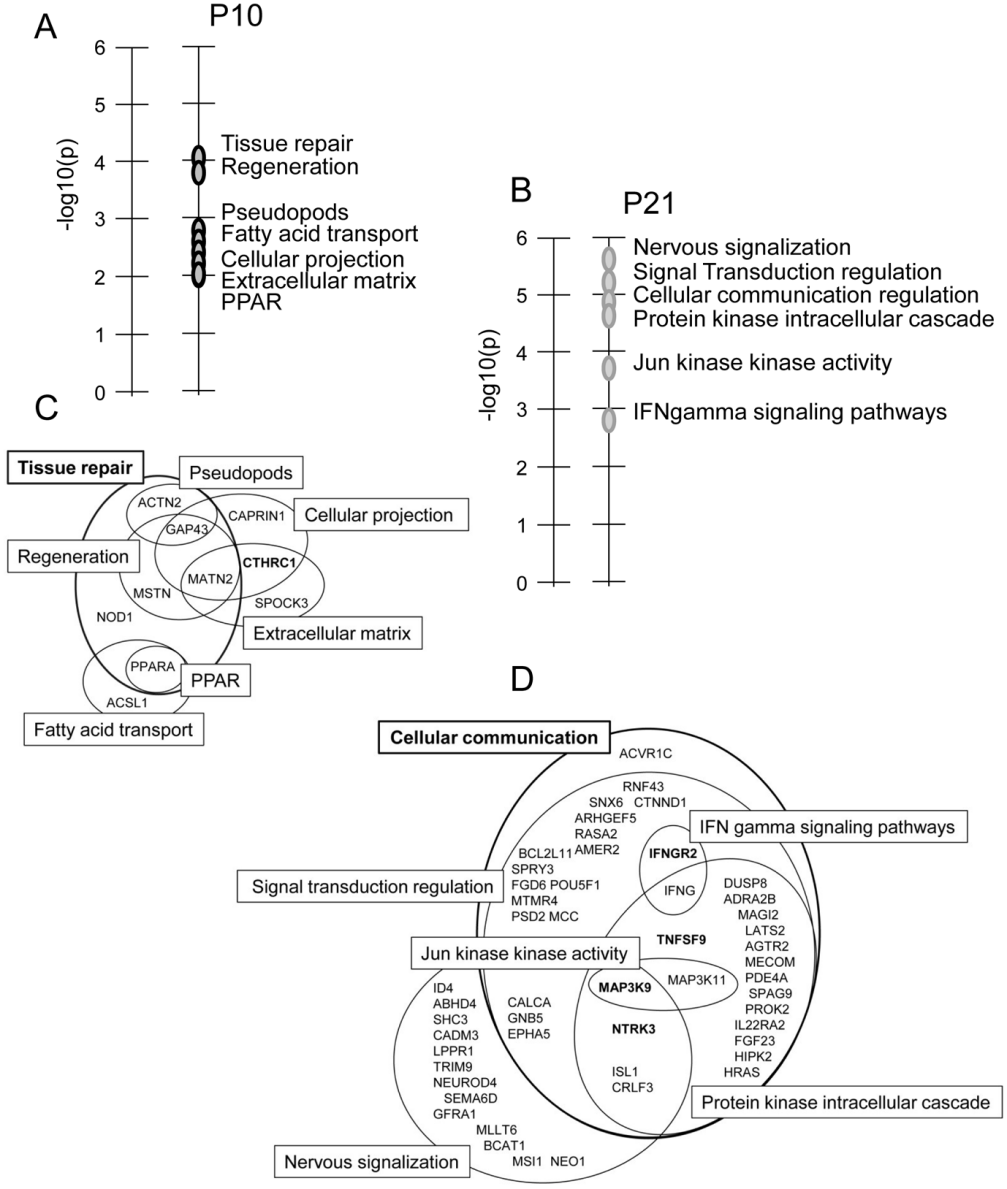
Gene ontology enrichment analysis of validated and predicted targets of miRNAs differentially expressed in rat lungs at P10 and P21 after LPD-induced IUGR. Comparison of the significantly deregulated genes at P10 and P21 with the validated and predicted targets of the deregulated miRNAs according to TarBase and Mirtarbase gave a new enriched list of 17 and 159 target genes at P10 and P21, respectively. A and B: The top significant pathways (*p* <0.01) linked to these genes at P10 and P21, respectively, were annotated according to Genomatix GePS. The vertical axis corresponds to enrichment score, which equals [-log10(*p* value)] and represents the significance level of pathways. C and D: Venn diagram showing the extent, at p10 and P21, respectively, of overlap between the predicted targets genes and genes identified in the literature as being implicated in these GO pathways.

We next extracted the predicted and experimentally observed gene targets for the 13 differentially expressed miRNAs from online miRNA databases. From this list of genes, we selected those shown on microarrays to be deregulated during alveolarization after LPD-induced IUGR [16]. At P10, 17 deregulated genes were predicted targets of our deregulated miRNAs, with 2 experimentally validated targets including CTHRC1 (Table 2 and S2 Table) and at P21, 159 deregulated genes were predicted targets, with 58 experimentally validated targets (Table 2 and S2 Table). This analysis featured E2F3, involved in cell cycle regulation, as it is a experimentally validated target of 5 of the 13 deregulated miRNAs. Of note, E2F3 is a target of miRNAs that are downregulated at P10 and upregulated at P21. GO analysis of these putative target genes revealed enrichment of genes (*p* < 0.01) related to “tissue repair”, “regeneration”, “pseudopods”, “Fatty acid transport”, “Cellular projection”, “extracellular matrix”, and “PPAR pathway” at P10 (Fig 3) and to “neuronal signalization”, “signal transduction regulation”, “cellular communication regulation”, “protein kinase intracellular cascade”, “Jun kinase kinase activity”, and “IFNgamma signaling pathways” at P21 (Fig 3). We next constructed Venn diagram to determine the extent of overlap between the predicted targets genes in our study and genes identified in the literature as being implicated in these GO pathways. At P10, a total of 10/17 predicted genes were identify in this analysis, including 6 genes that had been implicated in tissue repair and are shared among regeneration, pseudopods, and PPAR pathways. Four additional genes related to tissue repair singled out cellular projection, extracellular matrix, and fatty acid transports pathways (Fig 3), with the gene CHTRC1, an experimentally validated target at P10 being implicated in both cellular projection and extracellular matrix. At P21, 51/158 genes were clustered, including 38 genes that correspond to cellular communication and are shared among signal transduction regulation, IFN gamma signaling pathways, protein kinase cascade and Jun kinase kinase activity pathways, and 20 that have been implicated in nervous signalization (Fig 3). Among these 51 genes, we selected 4 target genes because no data were available on their expression in the rat developing lung: MAP3K9, shared within cellular communication, nervous signalization and kinase pathways; NTRK3, involved in cellular communication and nervous signalization; IFNGR2, in IFN gamma signaling; and TNFSF9, in protein kinase intracellular cascade.

**Table 2:** Experimentally validated targets (source: Tarbase and Mirtarbase) for the 13 significantly modulated miRNAs, sorted by Fold change in expression, during alveolarization in rats with low-protein diet-induced intrauterine growth restriction.

*Symbol*	*p* value	Fold change in expression	Detected at	P10 target genes	P21 target genes
miR-184	0.0184	0.18	P21	-	AP3D1
miR-127-3p	0.0041	0.40	P21	-	HECTD1
miR-128-3p	0.0082	0.44	P10	-	**E2F3, NTRK3**, ZFHX4, MUC13, GNB5, EFEMP2, MPP2, POU5F1, EYA4, SGPP1, TUB, NXT1, RASSF6
miR-378a-5p	0.0167	0.45	P21	-	ETNK1
miR-541-5p	0.0263	0.46	P21	-	-
miR-34c-5p	0.0232	0.49	P10	*-*	**E2F3**
miR-19b-3p	0.0273	2.10	P21	-	**E2F3**, ZFHX4, BCL2L11, EGLN3, ACPL2, , ID4, HECTD1, DLG5, NCOR2, SMARCA5, PDEA4, CHERP, PPTC7, STOX2, GLCE, SLC35F1
miR-434-3p	0.0036	2.18	P21	-	-
miR-449a-5p	0.0009	2.19	P21	*-*	**E2F3**
miR-1839-5p	0.0144	2.35	P21	*-*	*-*
miR-451-5p	0.0469	2.40	P21	-	-
miR-23b-5p	0.002	2.57	P21	-	**MAP3K9**, MTMR4, CTNND1, MECOM, LYL1, PNPLA2,HRAS, NCOR2, R3HDM2, TRPC4AP, HIPK2
miR-30e-5p	0.0305	2.90	P21	CAPRIN1, **CTHRC1**	**E2F3, IFNGR2, TNFSF9**, XPOT, GLCE, MLXIP, CYB561, ABCA12, HIPK2, CCDC120, UNC119B, TMCC1, SPAG9, SMARCA5, DSCC1, CCDC43, CEP120, SLITRK5, DLG5, RASA2, ZNF629, SC2, ELAVL2, GFRA1, HAS2,

In bold: genes further tested by qRT-PCR

**Fig 4:**
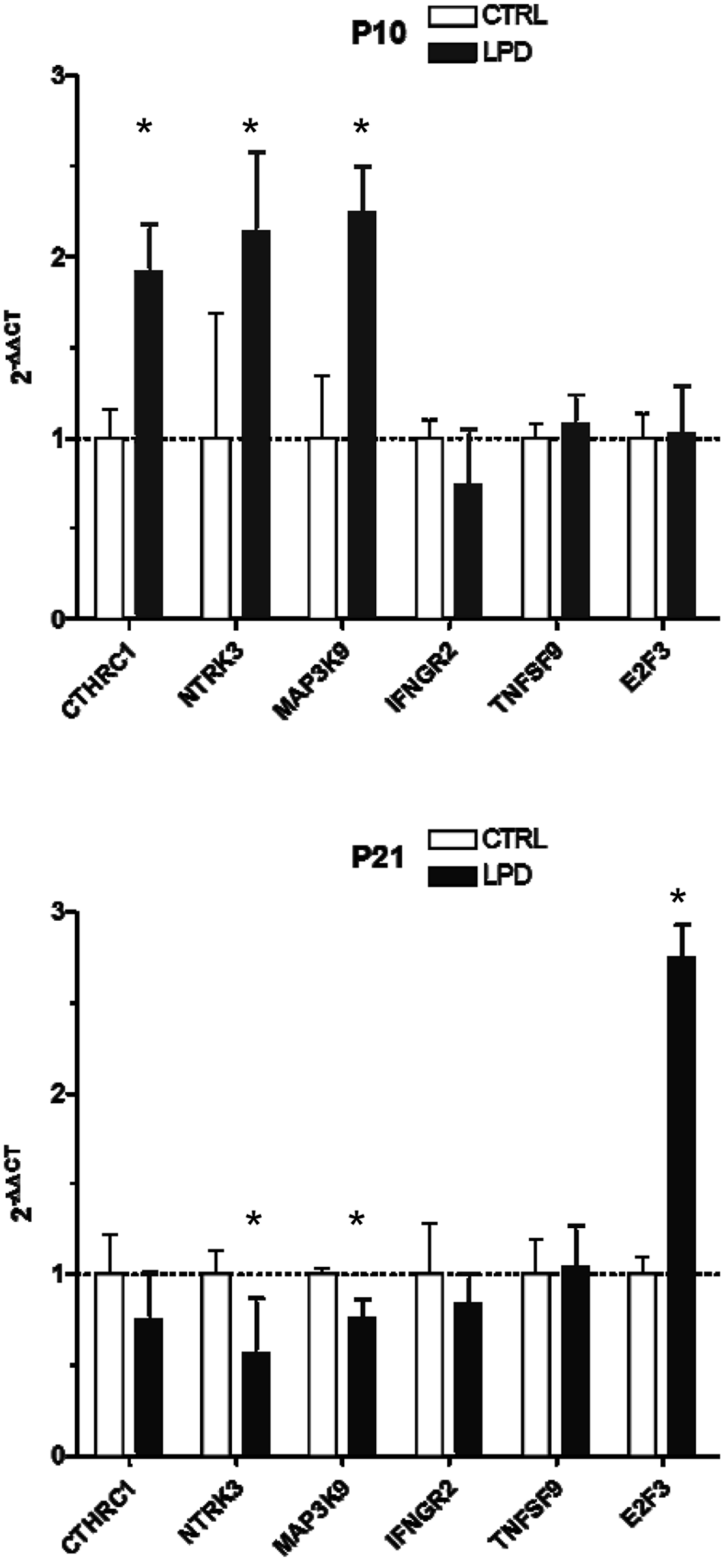
qRT-PCR analysis of validated mRNA targets of the miRNAs differentially expressed in lungs at P10 and P21 after LPD-induced IUGR. Five genes were selected from among the target genes according to their described function and quantified by qRT-PCR at P10 and P21. The relative expression was normalized to that of housekeeping genes (GAPDH and MMP16 [16]). Data are mean ± SEM of 5 animals/group. *, *p* < 0.05; two-tailed Mann-Whitney test.

We used qRT-PCR to examine the deregulation of E2F3, CTHRC1, MAP3K9, NTRK3, IFGNR2, and TNFSF9 genes at P10 and P21 in lungs during alveolarization after LPD-induced IUGR. Three genes, CTHRC1, NTRK3, and MAP3K9, showed significant upregulation at P10, with no change (CTHRC1) or significant downregulation at P21 (NTRK3 and MAP3K9) (Fig 4). E2F3 was significantly upregulated at P21 in LPD-induced IUGR lungs.

### Expression of E2F3 during alveolarization after LPD-induced IUGR

**Fig 5:**
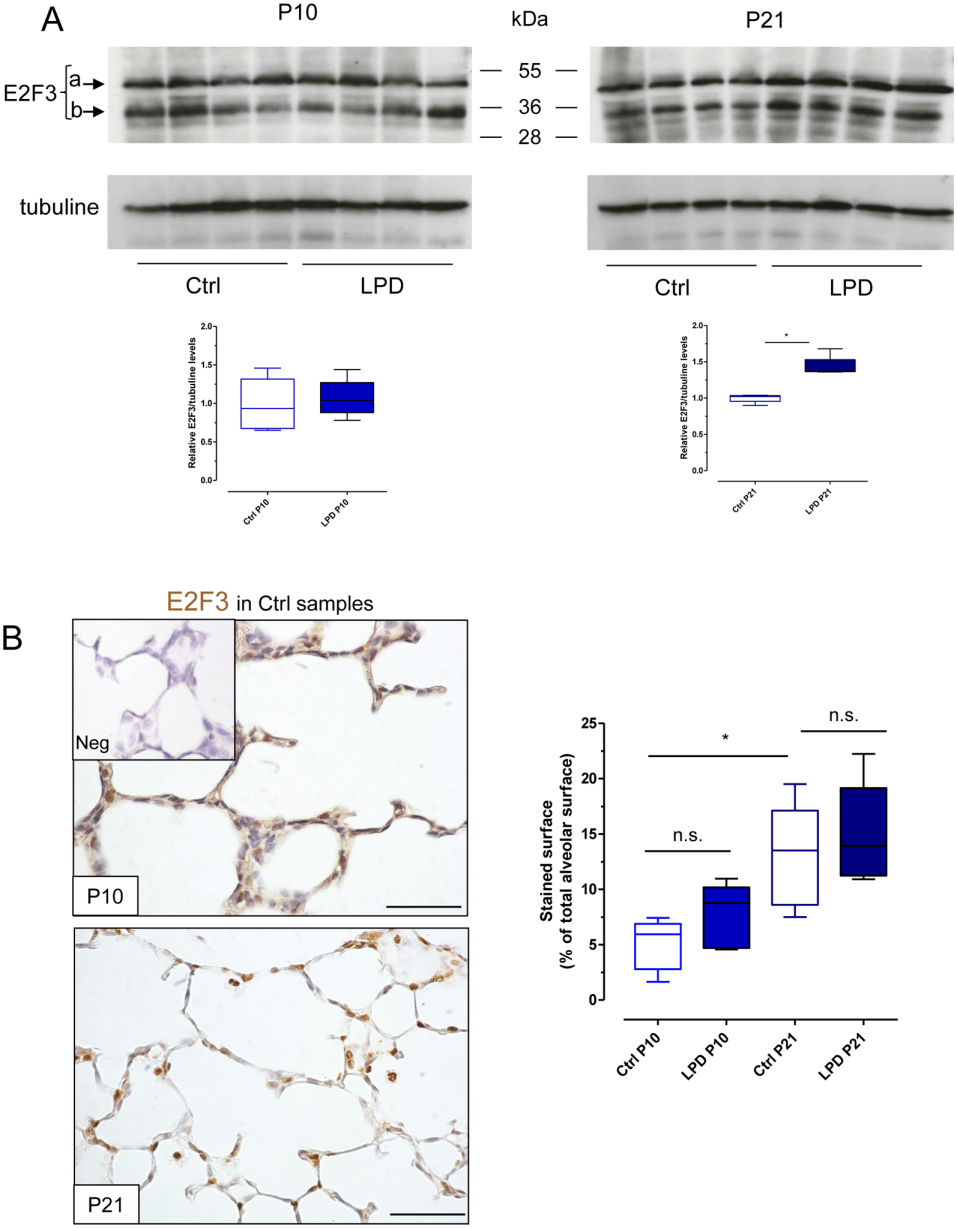
Western blot and immunohistochemistry analysis of E2F3 expression in lungs at P10 and P21 after LPD-induced IUGR. (A) Representative blots obtained with whole lung homogenates at P10 and P21 after LPD-induced IUGR or control-diet gestation. Beta-tubulin was the loading control. The two bands representing E2F3a and E2F3b were used for densitometric analysis shown below. Insert: box and whisker representation of median values for both E2F3a and E2F3b for 5 animals/group. *, *p* < 0.05; two-tailed Mann-Whitney test. (B) Representative images of E2F3 staining in the alveolar region at P10 and P21 for the control-diet gestation group. Insert: representative image of staining in normal rabbit serum; Neg: negative control. Scale bar, 100 μm. Quantification of staining within alveolar regions in LPD-induced IUGR and control groups at P10 or P21 shown on the right; box and whisker representation of median values for 5 animals/group. *, *p* < 0.05; two-tailed Mann-Whitney test.

We further attempted to establish the protein pattern of the significantly modulated genes by western blot assay and immunohistochemistry. No reproductive result was obtained for CTHRC1, NTRK3, and MAP3K9. E2F3 was present in whole-lung homogenates at P10 and P21, showing two forms corresponding to the size of E2F3a and E2F3b [17]. E2F3 protein level, measured as both E2F3a and E2F3b signals, was significantly increased at P21 in LPD-induced IUGR lungs (Fig 5). Immunohistochemistry revealed E2F3 expressed in alveolar regions (Fig 5), with a significant increase in E2F3 level in the alveoli surface from P10 to P21 in control samples, but no significant difference in level between LPD-induced IUGR and control lungs.

## Discussion

We previously showed that LPD-induced IUGR impaired alveolarization in the rat pup model [4]. Genome-wide microarray analysis of the alveolarization process in this model highlighted the perturbation of different gene networks at P10 and P21 [16], which raises the question of an upstream mechanism involved in this deregulation. We used global miRNA profiling analysis at these two key times of alveolarization to determine whether these epigenetic regulators of gene networks are specifically altered after LPD-induced IUGR. To our knowledge, this is the first miRNA analysis of IUGR-induced disorders of lung alveolarization. We found a significant deregulation, with more than twofold change in expression, of three mature miRNAs at P10 and 10 at P21.Target genes of these miRNAs were mainly related to “tissue repair” at P10 and to “cellular communication regulation” and “nervous signalization” at P21.

Dynamic changes in miRNAs have been reported during lung organogenesis [7, 18]. Accordingly, our unsupervised analysis of the global profile of miRNA expression in the rat whole lung at P10 and P21 significantly discriminated the two time points. Certainly, our selection of the two times is not sufficient to model alveolarization impairment in this IUGR model [19]. Both P10 and P21 represent snapshots of the dynamic, coordinated process of lung development and an integration of prenatal events and postnatal catch-up growth whereby molecular events of interest may precede phenotypic events. Nonetheless, in this model, thorough morphometric analysis showed a significant 30% decrease in alveolar surfaces at P10 and P21 with the LPD as compared with controls, with no significant difference observed at P4 [4]. The pups, with symmetric growth restriction (20% lower body weight) at P4 and P10, showed total catch-up growth at P21. Although important and mechanistic changes are likely present at earlier times than P10, we focused on these two later times of alveolarization, when gene deregulation was more pronounced [4, 16].

We next analyzed the two times separately and found a clear clustering of the two groups according to diet during gestation at P10, which was less obvious at P21. Nevertheless, we identified mature miRNAs with significant expression change at these two times after LPD-induced IUGR: 56 at P10 and 74 at P21, 7.7% and 10%, respectively, of the total rat mature miRNAs (728) on the Affymetrix 4.0 microarrays. Other teams investigated global deregulation of miRNAs in a rodent model of BPD [8, 9, 11, 12]. Despite the differences in animal models and in miRNA microarrays used and the fact that miRNA characterization is ongoing, Xing et al. and Zhang et al. also found upregulation of miR-449a-5p during the last stages of impaired alveolarization. This miRNA has been previously found highly expressed at the end of branching morphogenesis in the late pseudoglandular phase, throughout the canalicular phase, and during the differentiation of pulmonary epithelial ciliated cells [20, 21]. Among the other miRNAs found deregulated in our study, miR-34c-5p, miR-128-3p miR-184, miR127-3p, miR-30e-5p, and miR-23b-5p were also previously described as “tumour suppressors”[22–26], although many miRNA studies previously dealt with cancer or oncogenic proliferation states and the current analysis depended on previous reports.

To identify relevant pathways or functions during alveolarization that were linked to the aberrant patterns of miRNA expression, we then integrated the transcriptome data from our previous study of the same biological samples [16]. Ontology analysis of the common mRNAs revealed two distinct patterns of altered expression of genes at P10 and P21. This allowed for a new picture of the lung development stages that are disrupted after LPD-induced IUGR: extensive tissue remodeling at P10 and intra- and extracellular communication after lung morphogenesis being almost complete at P21. As previously noted, deregulation of the PPAR pathway, in relationship with tissue repair, may be a clue to the alveolarization impairment at P10 [16].

Of note, “nervous signalization” at P21 was among the top ontology pathways identified. This pathway has been poorly investigated during lung development. Axon development requires directional information via attraction or repulsion for the growth of the cone. Outgrowth of secondary septa during the formation of alveoli may involve similar mechanisms. Along this line, Vadivel et al. demonstrated that Semaphorin 3C, an axonal guidance cue of the Semaphorin family, is highly expressed during rat alveolar development, accelerates alveolar epithelial cell wound healing, and enhances endothelial cell networks in the hyperoxic model of BPD [27].

We further tested the expression of genes associated with these pathways that were not previously described during alveoli development. Several miRNAs have identical protein targets and present opposite deregulated expression, up or down, so one cannot predict target level change. We found significant deregulation of CTHRC1, NTRK3, and MAP3K9, with upregulation at P10 after LPD-induced IUGR, and repression at P21 for the two last genes. Notably, NTRK3 belongs to the neurotrophin receptor family, well known to be involved in regulating neuronal growth, differentiation, and survival [28, 29].

Furthermore, five deregulated miRNAs (miR-128-3p and miR-34c-5p, both downregulated at P10; miR-19b-3p, miR-449a-5p and miR-30e-5p, these three being upregulated at P21) have E2F3 in common as a target gene. E2F3 is a transcriptional activator that increases cell proliferation by promoting the G1/S transition and the initiation of DNA synthesis [30]. It is expressed in the lung epithelium, especially early in development, and interacts with Nkx2.1, well known to be involved in lung cell proliferation and survival [31]. We showed E2F3, present in the developing alveoli, upregulated at the mRNA level at P21 after LPD-induced IUGR, i.e. after downregulation at P10 of two miRNAs that are predicted to target E2F3. Data at the protein level were less revealing, with only a slight increase detected at P21. We also found differential modulation of the mRNA expression of CTHRC1, NTRK3, and MAP3K9 at P10 and P21. Unfortunately, we could not pursue the analysis of these three genes at the protein level because of the absence of reliable antibodies in our experimental conditions. Nonetheless, these results question the temporal link between the deregulated listed miRNAs and the expression of E2F3 and possible loop of regulation. Larger-scale studies at the protein level are required to establish causal links between miRNA deregulation and alterations of alveolarization.

Lung development is a coordinated action of growth and transcription factors, extracellular remodeling, cell differentiation, and physical forces. The classical experimental models of BPD based on oxygen toxicity and baro- and volo-trauma, such as hyperoxia and mechanical ventilation, were designed to mimic extrauterine injury to the immature lungs [32, 33]. Compelling evidence suggests that lung development can be markedly primed in utero. Especially, IUGR in animal models consistently confers impaired alveolar development [16, 34]. IUGR commonly results from maternal utero-placental insufficiency that occurs in association with vascular disorders such as preeclampsia. LPD in animal models mimics uteroplacental insufficiency and the impaired placental nutrient transport observed in IUGR. The degree of lung structural change and cellular dysfunction/repair with IUGR/LPD modeling remains to be clarified. Molecular markers, such as miRNAs, have the potential for personalizing the assessment of primary injury risk and treatment of repair pathways.

Other limitations to our study should be noted. The animal number per group was low yet sufficient to reveal specific miRNAs. However, this small sample does not allow for addressing sex-specific differences, because some pathways or regulation may differ by gender [35, 36]. As well, we analyzed whole lung samples, which hides discrete modifications within specific cell types in the developing alveoli. However, alveolarization is a coordinated process involving paracrine mechanisms between lung fibroblastic, epithelial, microvascular, and extracellular matrix components [37]. Our global analysis revealed the major changes during this process. Although we can draw no direct causal link between miRNA changes and inhibition of alveolar development from our current results, they are indicative of a network alteration of gene regulation and may serve as stable indication of evolution toward BPD. Additional mechanistic evidence is needed to fully evaluate how the miRNA levels are affected by diet during gestation or the causal relationship between specific miRNAs and putative targets.

To conclude, the pathogenesis of alveolarization impairment remains poorly understood. The increased incidence of BPD in preterm infants born with IUGR draws attention to a relationship between IUGR and BPD, and antenatal programming of lung development. Using a model of IUGR induced by protein restriction during gestation, we screened changes in expression of miRNAs during the alveolar septation phase in rat. miRNAs are indeed deregulated during alveolarization after LPD-induced IUGR, which suggests *in utero* epigenetic programming of BPD with IUGR. MiRNAs are highly conserved among species [38]. They are present in tissue and in circulating blood [6]. The miRNAs we detected could be valuable biomarkers to detect very early lung alveolarization disorder in human preterm babies. The analysis of the aberrant patterns of miRNA expression may also shed light on previously unknown pathways and genes with altered expression.

## Supporting information

S1 Table Detailed data of the 56 and 74 miRNAs modulated at P10 and P21. (XLSX)

S2 Table Predicted and experimentally validated targets of miRNAs modulated at P10 and P21. (XLSX)
